# Supplementary material for: Influence of age and sex on winter site fidelity of sanderlings Calidris alba
Source: PeerJ. 2016 Sep 28;4:e2517. doi: 10.7717/peerj.2517 (PMC5045889; doi:10.7717/peerj.2517)
Supplement: Appendix S1 [file peerj-04-2517-s002.pdf]

Appendix

*Biometric sexing of sanderlings (*Calidris alba*)*

Simple and reliable sexing methods based on biometrics are useful when invasive techniques, such as blood sampling to obtain DNA for molecular sexing, cannot be applied. We investigated the accuracy of a sexing method for sanderlings *Calidris alba* that exclusively relies on biometric data.

Methods

A total of 990 sanderlings were captured in their wintering areas in Mauritania and Ghana between 2004 and 2009. The bill length, tarsus length and total head length were measured to the nearest 0.1 mm using a sliding calliper. Wing length and tarsus and toe were measured using a stopped ruler to the nearest 1 mm. DNA was extracted from a small blood sample (~50 µL) taken from the brachial vein of each individual and used to molecularly sex each individual using methods as described in detail by Reneerkens et al. (2014).

We used generalized linear models with binomial errors and a logit-link function to examine the relationship between the molecular sex, wing length, tarsus length, tarsus-toe length, bill length and total head length and their interactions, with sex as the binary response variable. The significance of the model components was tested in a stepwise manner by testing the deviance of the likelihoods of full and reduced models, excluding the component of interest (Zuur 2008). This statistical method gives estimates of the parameters $ß_i$ that are required to predict the sex of an individual. Collinearity of all the variables was estimated with the variance inflation factor (VIF; Quinn & Keough 2002). The statistical analyses were conducted using R (version 2.13.0, R Development Core Team 2012).

Results

Of the 990 sanderlings that were sexed using molecular methods, 549 were males and 441 females. Females were on average significantly larger than males (Table S1) but all biometric measurements overlapped to some extent (Table S1). Because variance inflation factors (VIF) for wing (1.37), tarsus (1.68), tarsus-toe (2.86), bill (3.28) and total head (3.86) were relatively small, there was no severe impact of multicollinearity.

Four measurements, including bill length (B), head length (H), wing length (W) and tarsus length (T), contributed significantly to the determination of sex. Adding tarsus-toe (TT) did not improve the results and even resulted in a higher chance of a wrong assignment. The equation based on the estimated values for the intercept and the corresponding $ß_i$ is:

$$P_{(male)} = \frac{e^{(-6486+277.0B+128.4H+50.51W-0.3635T-5.456B.H-2.148B.W-0.9946H.W+0.0423B.H.W)}}{1+e^{(-6486+277.0B+128.4H+50.51W-0.3635T-5.456B.H-2.148B.W-0.9946H.W+0.0423B.H.W)}}$$

The model correctly predicted the sex of 83.7% individuals.

Tables

Table S1. Comparison of all biometric measurements for male and female sanderlings that were molecular sexed. We present the number (n) of males and females used for analysis, the mean (±SD) and range for each biometric and the t statistic (t) for the comparison of averages between sexes.

	Males (n=549)		Females (n=441)		
	mean	range	mean	range	t
Wing (mm)	124.9±2.9	116-138	127.9±3.1	117-137	15.6***
Tarsus toe (mm)	44.1±1.7	40-49	45.1±1.7	40-49	8.7***
Total head (mm)	49.0±1.3	44.2-53.0	51.3±1.6	41.1-56.6	24.4***
Tarsus (mm)	24.5±0.9	21.1-28.0	25.6±1.1	22.2-28.8	17.0***
Bill (mm)	23.9±1.1	20.2-27.5	25.7±1.2	21.8-29.1	24.3***

*** P <0.001

Table S2. Model comparisons of full and reduced models. Deviance and probability are due to the last component added to the models. AIC (Akaike Information Criterion) reflects the overall fit of the model while correcting for the number of parameters. Lower values for deviance and AIC indicate a better fit. The most parsimonious model is indicated in bold. For each model we present degrees of freedom (DF), deviance, P value and AIC, as well as the proportion of molecularly sexed sanderlings that the model correctly sexed using biometric information. B: Bill length, H: head length, W: wing length, T: tarsus length, TT: tarsus toe length.

Model	Df	Deviance	*P*	AIC	Correctly sexed (%)
Sex = B · H · W · T · TT	958	771.73	0.026	794.73	83.6

**Sex = B · H · W · T**	**781**	**768.61**	**$5.2 \cdot 10^{-04}$**	**786.61**	**83.7**
Sex = B · H · W	982	780.72	$9.2 \cdot 10^{-09}$	796.72	83.6
Sex = B · H	986	823.97	$2.2 \cdot 10^{-16}$	831.97	83.4
Sex = B	988	897.25	$2.2 \cdot 10^{-16}$	901.25	79.7

References

Quinn G. P. & M. J. Keough 2002. Experimental design and data analysis for biologists. Cambridge University Press, Cambridge, UK.

Prater A.J., Marchant J.H. & Vuorinen J. 1977. Guide to the identification and ageing of Holarctic waders BTO Guide 17. British Trust for Ornithology, Tring

R Core Team. 2012. R: A language and environment for statistical computing. R foundation for Statistical Computing, Vienna.

Reneerkens, J., van Veelen, P., van der Velde, M.,Luttikhuizen, P. & Piersma T. 2014. Within-population variation in mating system and parental care patterns in the Sanderling (*Calidris alba*) in northeast Greenland. *The Auk: Ornithological Advances* 131: 235-247.

Zuur, A.F. 2008. Mixed effects models and extensions in ecology with R. Springer Press
